# Editorial: Celebrating the work of Prof. C. N. R. Rao: from solid state to materials chemistry

**DOI:** 10.3389/fchem.2024.1501765

**Published:** 2024-10-25

**Authors:** Cyril O. Ehi-Eromosele, Yashonath Subramanian

**Affiliations:** ^1^ Department of Chemistry, Covenant University, Ota, Nigeria; ^2^ Solid State and Structural Chemistry Unit, Indian Institute of Science, Bangalore, India

**Keywords:** Prof. C. N. R. Rao, solid state chemistry, materials chemistry, 2-D materials, nanomaterials


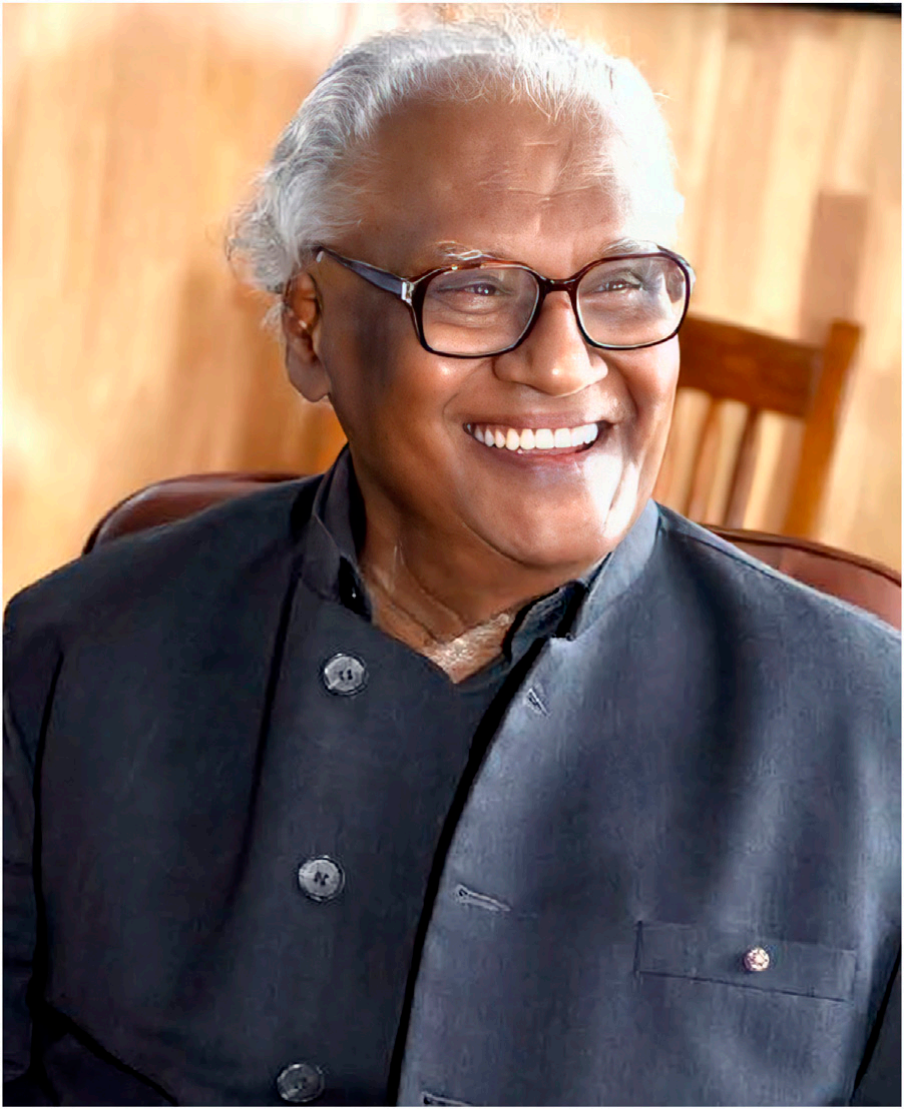


This Research Topic is dedicated to the extraordinary scientific contributions of Prof. C.N.R. Rao to the field of Chemistry generally and to solid state/material chemistry in particular. Prof. Rao is the Founder President of Jawaharlal Nehru Centre for Advanced Scientific Research (JNCASR), an institution he is still associated with leading an impressively large chemistry and physics of materials group. Following his early researches in spectroscopic methods, Prof. Rao has gone on to make immense contributions to solid state chemistry, chemistry of oxides, low-dimensional materials, nano and hybrid materials, material synthesis (mostly solid-state methods) and recently, materials for energy and environmental applications, among many others. Over these years, spanning about 7 decades, he has authored about 1800 publications, more than 56 books, garnered an impressive Scopus citation record (over 103000 citations and an h-index of 150), and has mentored many young scientists including the Research Topic Editors.

Beyond the confines of the laboratory, Prof. Rao has played major roles in policy, advocacy and development of science both in India and globally, mostly in the less developed world. He has an impressive track record of building many scientific institutions in India such as JNCASR, several nano institutes (under the India’s Nano mission), Indian Institutes of Science Education and Research, etc. He was also the Director of the Indian Institute of Science from 1984 for a decade. Also, Prof. Rao has been an advocate of knowledge equality amongst the world’s population. In fulfilling this passion, he was the President of the Third World Academy of Sciences (TWAS). TWAS has helped to train hundredths of young scientists from the developing world and has also facilitated the establishment of science academies, stimulating science in many of these science-disadvantage countries. In addition, he also served as President of the International Union of Pure and Applied Chemistry (IUPAC). An important part of his profile is that Prof. Rao have been able to accomplish these in less than the most favorable circumstances demonstrating his sheer determination and commitment. On the humane side, Prof. Rao has helped many bright and deserving persons to find a job, get an award or simply by appreciating his/her work and inviting them to meetings organized by him. Not infrequently, he has helped people in distress due to health issues, to find qualified and excellent doctors.

This Research Topic is a celebration of the interactions between some established and early-career materials chemists who have either directly collaborated or have been inspired by the works of Prof. Rao. The Research Topic covers a range of topics including the revelation of a new structural phase for high-pressured and high temperature orthorhombic R_2_BaCuO_5_ (R = Sm and Eu), the synthesis of a hybrid MoS_2_-carbon material used as high-performance electrode material in supercapacitor, the application of a new ceramic thermistor material with good negative temperature coefficient (NTC) at high and wide temperature ranges, and solar enhanced oxygen evolution reaction with transition metal telluride.

In the article, “*High-pressure synthesis and magnetic properties of tetragonal R*_*2*_ *BaCuO*_*5*_ *(R = Sm and Eu)*,” by Mishra et al., the authors used high-pressure synthesis to convert R_2_BaCuO_5_ (R = Sm and Eu) normally with an orthorhombic phase to a novel tetragonal phase with varying magnetic properties. The magnetic analyses revealed that Eu_2_BaCuO_5_ in the tetragonal phase is a promising magnetocaloric material. These materials are of interest due to their applications in innovative cooling technologies. Magnetocaloric refrigeration systems are currently at various stages of commercial development. Overall, the study demonstrates the potentials of using high-pressure synthesis to obtain novel materials.

Khandare et al. in the article, “*MoS*_*2*_ *nanobelts-carbon hybrid material for supercapacitor applications,*” synthesized a low-cost hybrid MoS_2_ nanobelts-carbon electrode material possessing a stable cycle life and high-specific capacitance in a supercapacitor. This high-performance electrode material also has potentials in other electrochemical storage systems, other than supercapacitors.

The research paper, “*High-stability solid solution perovskite (1-x) Bi*_*0.2*_ *Sr*_*0.5*_ *La*_*0.3*_ *TiO*_*3*_ *-xLaMnO*_*3*_ *(0.05≤ × ≤0.2) for wide-temperature NTC thermistors*” by Liu et al. presented a new high temperature NTC thermistor material with perovskite structure. Unlike other known NTC materials, this solid solution ceramic materials have low sintering temperature, stable structure and display good electrical characteristics over a wide temperature range (25°C–600°C). The exceptional conduction mechanism of this thermistor materials was also revealed which could facilitate the production of other excellent NTC ceramic thermistors.

“*Solar enhanced oxygen evolution reaction with transition metal telluride*” is a research paper by Singh et al. Herein, nickel telluride showed attractive catalytic activity in a solar-coupled electrochemical oxygen evolution reaction (OER) recording enhanced photo response, excellent light harvesting ability, and increased interfacial kinetics of charge separation. NiTe which is a non-precious transition metal with low operating cost is a highly efficient electrocatalyst for light activated water splitting leading to very low OER overpotential.

